# Sex differences in behavior, cognitive, and physiological recovery following methamphetamine administration

**DOI:** 10.1007/s00213-024-06638-1

**Published:** 2024-07-02

**Authors:** Monserrat Armenta-Resendiz, Jordan S. Carter, Zachariah Hunter, Makoto Taniguchi, Carmela M. Reichel, Antonieta Lavin

**Affiliations:** Department of Neuroscience, MUSC, 173 Ashley Ave 403BSB, Charleston, SC 29425 USA

**Keywords:** Hypofrontality, Methamphetamine, GABA, sex differences

## Abstract

Intact executive functions are required for proper performance of cognitive tasks and relies on balance of excitatory and inhibitory (E/I) transmission in the medial prefrontal cortex (mPFC). Hypofrontality is a state of decreased activity in the mPFC and is seen in several neuropsychiatric conditions, including substance use disorders. People who chronically use methamphetamine (meth) develop hypofrontality and concurrent changes in cognitive processing across several domains. Despite the fact that there are sex difference in substance use disorders, few studies have considered sex as a biological variable regarding meth-mediated hypoactivity in mPFC and concurrent cognitive deficits. Hypofrontality along with changes in cognition are emulated in rodent models following repeated meth administration. Here, we used a meth sensitization regimen to study sex differences in a Temporal Order Memory (TOM) task following short (7 days) or prolonged (28 days) periods of abstinence. GABAergic transmission, GABAA receptor (GABA_A_R) and GABA Transporter (GAT) mRNA expression in the mPFC were evaluated with patch-clamp recordings and RT-qPCR, respectively. Both sexes sensitized to the locomotor activating effects of meth, with the effect persisting in females. After short abstinence, males and females had impaired TOM and increased GABAergic transmission. Female rats recovered from these changes after prolonged abstinence, whereas male rats showed enduring changes. In general, meth appears to elicit an overall decrease in GABA_A_R expression after short abstinence; whereas GABA transporters are decreased in meth female rats after prolonged abstinence. These results show sex differences in the long-term effects of repeated meth exposure and suggest that females have neuroprotective mechanisms that alleviate some of the meth-mediated cognitive deficits.

**Supplementary Information:**

The online version contains supplementary material available at 10.1007/s00213-024-06638-1.

## Introduction

Chronic methamphetamine (meth) use disorder (MUD) is a severe health problem that can lead to impairments in executive function across multiple cognitive domains. Importantly, correct cognitive performance rely on intact function of the medial prefrontal cortex (mPFC). There are sex differences in MUD with women starting to consume meth at earlier age and rapidly graduating to regular use (Brecht et al. 2004; Yen and Yang 2005; Wu et al. 2007). Women also maintain a stronger dependence or loyalty to the drug when compared to men (Hartwell et al. 2016; Rawson et al. 2002, 2005; Su et al. 2017; Yimsaard et al. 2018) and are more receptive and responsive to treatment for MUD (Liu 2008; Price et al. 2011; Ray et al. 2015). Despite these sex differences, historic underrepresentation of women in clinical samples has led to little existing research directly comparing cognitive function in men and women with MUD (Martin et al. 2021). Those that do include sex as a biological variable show that men score lower than women on cognitive tests of behavioral flexibility (van der Plas et al. 2009) and verbal memory (King et al. 2010).

In both human clinical (Okita et al. 2016; Simon et al. 2010; Dean et al. 2011; van Nunen et al. 2021; London et al. 2015; Baicy and London 2007; Monterosso et al. 2007; Payer et al. 2008; Nestor et al. 2023 and preclinical animal models (Bernheim et al. 2016; Armenta-Resendiz et al. 2022), changes in cognitive processes following repeated meth exposure lead to deficits in episodic and working memory, involving dysregulation of the mPFC. During acute meth intoxication the mPFC is activated, but during abstinence following prolonged use the mPFC is hypoactive (Goldstein and Volkow 2011; Bisagno et al., 2016. Cortical hypoactivity refers to an imbalance in the normal excitation/inhibition neurotransmission (E/I) ratio in the mPFC. We posit that alterations in the E/I ratio may underlie hypofrontality and the ensuing cognitive sequalae found in various neuropsychiatric disorders, such as schizophrenia (Sohal and Rubenstein 2019; Lopatina et al., 2019), anxiety (Park et al., 2016), substance use disorder (Goldstein and Volkow, 2011; Goldstein et al. 2002; Volkow 2009, and depression (Kawabata et al. 2022; Ohta et al. 2008). Individuals who use meth chronically develop hypofrontality (Kim et al., 2005; Kim et al. 2009) with concurrent deficits in verbal memory, working memory, and executive function (King et al. 2010). Similarly, repeated meth administration in rodents also elicits hypofrontality and memory deficits (Armenta-Resendiz et al. 2022; Nagai et al. 2007; Lee et al. 2011). Despite the presence of sex differences in some cognitive measures, only minor effects of meth were reported in animals, indicating that both male and female rodents are equally susceptible to meth-induced cognitive deficits (Acevedo et al. 2007; Siegel et al. 2010; Reichel et al. 2012; Ghazvini et al. 2016; Hankosky et al. 2018; Davis et al. 2022). However, it is unknown whether there are sex differences in the persistence of this cognitive impairment during abstinence.

We have previously reported baseline sex differences in spontaneous excitatory activity in the mPFC, with males having increased excitability relative to females following meth self-administration and 7 days abstinence (Pena-Bravo et al. 2019. We also reported increased GABAergic transmission in the mPFC associated with changes in E/I ratio and cognitive deficits after repeated meth treatment in male rats after 7 days of abstinence (Armenta-Resendiz et al. 2022). In the current study, using a repeated meth administration paradigm as a tool to model cognitive deficits found in meth use, we investigate the mechanisms underlying meth-mediated sex differences following a short abstinence (7 days) and a prolonged abstinence (28 days) period. In a supplemental experiment using ovariectomized (OVX) females, we also began to characterize the contributions of gonadal sex hormones to these sex differences.

## Materials and methods

### Animals

**Fig. 1:**
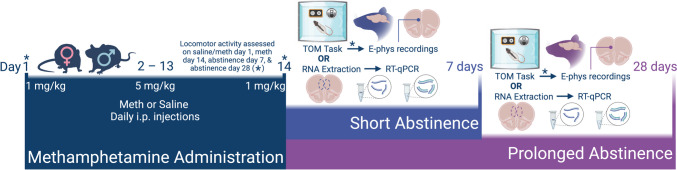
Experimental Timeline. Male and female rats received daily injections of saline or meth (1 mg/kg on days 1 and 14, 5 mg/kg on days 2–13) for 14 days, follow by 7 (short) or 28 (prolonged) days of abstinence. Locomotor activity was assessed following meth administration on days 1 and 14, and on abstinence days 7 and 28. At each abstinence timepoint, a subset of rats completed the TOM task followed by electrophysiology recordings in the mPFC. A separate group of rats had their mPFC processed for RT-qPCR analysis

The experimental timelines are shown in Fig. 1. The studies used age matched adult male and female Long Evans rats (2 months old) from our breeding colony. Males and female rats weighed between 250–350 g. OVX female rats (Charles River Laboratories) were obtained at 60–90 days of age. OVX experiment data and analyses are included in the Supplement. Rats were pair housed under 12 light/12 dark hour cycle, with access to food and water ad libitum. All experiments were performed during the light cycle and in accordance with the National Institutes of Health guidelines for the care and use of laboratory animals. Procedures are approved by the Institutional Animal Care and Use Committee (IACUC) of the Medical University of South Carolina.

### Meth administration and locomotor activity assessment

Methamphetamine hydrochloride (NIH, NIDA Drug Supply) was dissolved in sterile saline and administered intraperitoneally (i.p.). Rats were assigned to either the meth or saline group, with daily injections according to the following schedule (Fig. 1): Day 1 rats received a single i.p. injection 1 mg/kg meth or saline. Days 2 to 13, rats received 5 mg/kg meth or saline, then, on Day 14, rats received meth (1 mg/kg) or saline. Following meth or saline treatment, rats received 7 days (herein termed short abstinence, A7) or 28 days (herein termed prolonged abstinence, A28) of home cage abstinence, during which animals are left undisturbed (Armenta-Resendiz et al. 2022; Wearne et al. 2016).

On meth administration days 1 and 14, following 1 mg/kg meth, locomotor activity was assessed. Rats underwent a locomotor test in clear acrylic chambers (40 × 40 × 30 cm) equipped with Digiscan monitors (AccuScan Instruments Inc., Columbus, OH, USA). Each chamber contained a 16 × 16 photobeam array for the x and y axes and 16 photobeams for the z axis. Photobeam breaks were detected by a Digiscan analyzer and horizontal activity was recorded by DigiPro software (Version 1.4). Rats were also tested for locomotor activity following the TOM test on abstinence day 7 and 28 with 1 mg/kg meth.

### Temporal order memory

**Fig. 2:**
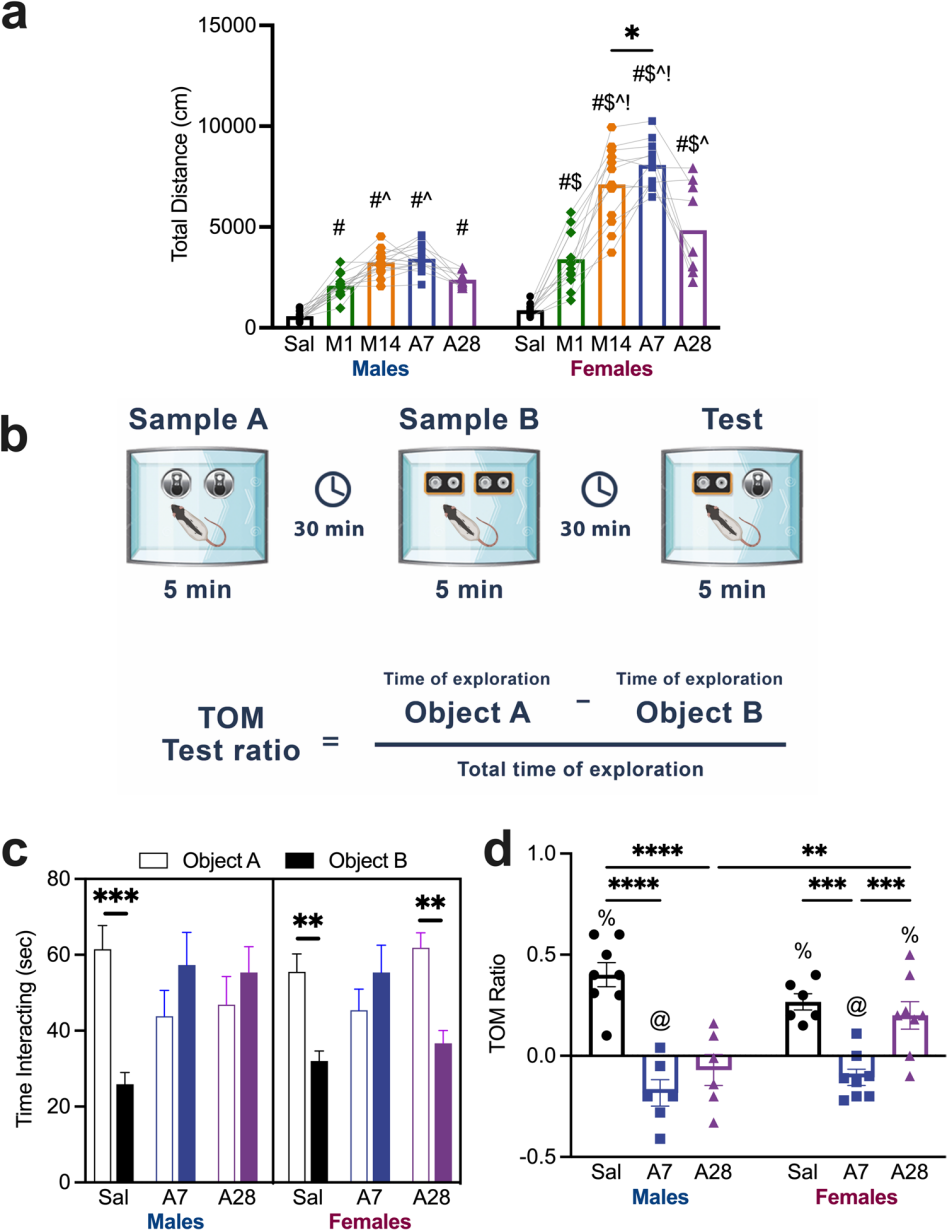
Meth induces locomotor sensitization and disrupts temporal order memory during abstinence in a sex-specific fashion. **A**: Distance traveled (cm) following meth exposure. Males and females increase locomotor activity following meth exposure, but do so in a sex-specific pattern (Holm-Šídák’s post-hocs: # p < 0.05 vs saline; $ p < 0.05 vs males; ^ p < 0.05 vs M1, ! p < 0.05 vs A28, * p < 0.05 between M14 and A7). **B**: Schematic of TOM task and calculation of TOM ratio. **C**: Time interacting (sec) with Object A (first object) and Object B (second object) during TOM test. Saline males and females spent more time with Object A. After 7 days of abstinence from meth, males and females spent similar time with both objects. After 28 days, males maintained this pattern, but females spent more time with Object A (Holm-Šídák’s post-hocs; ** p < 0.01, *** p < 0.001). **D**: TOM discrimination ratios. Saline males and females preferred Object A (%, p < 0.05 preference for Object A vs chance). On A7, both sexes preferred Object B (@, p < 0.05 preference for Object B vs chance). On A28, males exhibited no preference, but females again preferred Object A. Males do not recover TOM performance after 28 days of abstinence, while females do (Holm-Šídák’s post-hocs; ** p < 0.01, *** p < 0.001, **** p < 0.0001). All data are shown as mean ± SEM with individual data points. Abbreviations: Sal: Saline, M1: Meth Day 1, M14: Meth Day 14; A7: Abstinence Day 7; A28: Abstinence Day 28, TOM: Temporal Order Memory

To assess cognitive function, we used the TOM task (Fig. 2B), which relies on the mPFC and perirhinal cortices (Barker et al. 2007; Barker and Warburton 2011) for proper execution and is impaired by repeated meth exposure Armenta-Resendiz et al. 2022). Beginning on day 4 or 25 of abstinence, rats were habituated for 2 consecutive days to a rectangular plastic arena (45 × 35 × 40 cm) for 5 min without objects. Sampling and test trials were all conducted on day 7 or 28 of abstinence. Sampling occurred in two trials with counterbalanced objects. During the first sample trial, a rat was placed into the arena and allowed to explore two identical objects (Object A; cylindrical black pencil holders, 11.5 cm high, 28 cm diameter) for 5 min. Then, the rat was returned to its home cage for 30 min. The second sample trial consists of 5 min exploration using a different pair of identical objects (Object B; 5 V batteries 6.3 × 6.3 cm, high: 9.5 cm). The rat returns to its home cage for 30 min. Finally, during the test, the rat was placed into the arena with one object from each sample (i.e., Objects A and B) for 5 min. The identities of the objects were counterbalanced, and rats demonstrated no preference for either object at baseline. The tests were recorded, and videos were analyzed post hoc by observers blinded to the order of the object presentation and drug group of the rat. The criteria for considering behavioral exploration of the object are sniffing and physically interacting with the object, but not leaning on it. Based on the innate tendency for rats to interact with novel objects, rats spend more time with the first object (object A) on test. TOM is inferred by a preference for object A because the rat more recently interacted with object B. Time spent with object A and B were recorded and a discrimination ratio was calculated with the following formula: time spent exploring object A – B / total time spent exploring both objects (Fig. 2). A positive value indicates a preference for the first object (i.e., primacy effect); a value close to 0 indicates chance exploration of both objects; a negative value indicates a preference for the second object (i.e., recency effect). The bedding in the arena was changed between rats, objects were cleaned between rats, and male and female rats were assessed on separate days.

### Brain slice preparation

Twenty-four hours after the TOM test, animals (7 or 28 days of abstinence) were deeply anesthetized using isoflurane (Minrad Inc., Orchard Park, NY, USA), rapidly decapitated, and their brain was extracted. Coronal slices 300 µM thick containing the PFC were obtained with a vibratome (Leica VT 1200 s, Leica Microsystems, Buffalo Grove, IL, USA) in ice-cold artificial cerebrospinal fluid (aCSF, containing: 200 mM sucrose, 1.9 mM KCL, 1.2 mM NaHPO_4_, 33 mM NaHCO_3_, 6 mM MgCl_2_, 10 mM D-glucose, and 0.4 mM ascorbic acid (pH: 7.3–7.4, 305–310 mOsm). Slices were incubated in a hot bath at 31 °C for 1 h before recordings, with a physiological solution containing: 120 mM NaCl, 2.5 mM KCl, 1.25 mM NaH_2_PO_4_, 25 mM NaHCO_3_, 4 mM MgCl_2_, 1 mM CaCl_2_, 10 mM D-glucose, and 0.4 mM ascorbic acid, oxygenated 95% O_2_ / 5% CO_2_ (pH: 7.3 – 7.4, 305–310 mOsm).

### Electrophysiological recordings

Electrophysiological recordings were performed as described previously (Armenta-Resendiz et al. 2022). Pyramidal neurons located in layers V-VI of the mPFC were recorded using whole-cell patch clamp techniques. Neurons were identified using infrared-differential interference contrast optics and video microscopy. Signals were recorded using an Axoclamp 2B (Molecular Devices) low-pass filtered at 3 kHz and digitized at 10 kHz. Data acquisition used Axograph-software (Axograph J. Clements). For voltage-clamp recordings, electrodes (3–5 MΩ resistance in situ) were fill with a solution containing: 130 mM CsCl, 10 mM HEPES, 2 mM MgCl_2_, 0.5 mM EGTA, 2 mM Na_2_ATP, 0.3 mM Na-GTP, 2 mM QX-314, 10 mM phosphocreatine, (pH = 7.2–7.3, 270–280 mOsm). The membrane potential was clamped at -70 mV. Series resistance (Rs) was monitored throughout the experiment using a -5 mV pulse (50 ms duration), and recordings that exhibit changes in baseline > 30% were discarded. GABAergic-mediated events were pharmacologically isolated by adding CNQX (10 µM) and AP-V (50 µM) to the aCSF recording solution. Spontaneous inhibitory post-synaptic current (sIPSC) recordings comprised five sweeps/10 s. Evoked inhibitory post-synaptic currents (eIPSCs) and paired-pulse (PP) recordings consisted of 8–10 repetitions at 0.5 Hz using the minimum current needed to evoke a response. The frequency and amplitude of sIPSCs, the amplitude of eIPSCs and paired-pulse ratio were analyzed using MiniAnalysis® software (Synaptosoft, Fort Lee, NJ USA**)**. The average stimulation current was 94.1 ± 97.4 pA. Synaptic currents were evoked using bipolar theta capillaries filled with saline solution placed in the mPFC around 300 µm from the patched neuron.

### RNΑ extraction

Separate groups of rats were used for the RNA experiments. A total of 36 rats received either meth or saline (detailed in the meth administration section). Male and female rats were distributed across 3 groups: saline, abstinence 7 days, or abstinence 28 days (n = 6/group). After meth or saline exposure, rats were euthanized via rapid decapitation on either the 7th or 28th day of abstinence. The mPFC was dissected from coronal sections (~ 25 mg of tissue), retrieved using 1 mm brain matrices over ice, and immediately frozen with dry ice before storing at -80 °C until RNA extraction.

Rnase*Zap*™ (Invitrogen/Thermo Fisher Scientific, Waltham, MA) was used throughout the RNA extraction and purification process to prevent any RNase activity and subsequent RNA degradation. Prior to RNA extraction, mPFC tissue samples were thawed and homogenized using 200 µL QIAZOL® lysis reagent (Qiagen, Limburg, Germany), plastic pestles, and a cordless motor (VWR, Radnor, PA). New pestles were used between samples to prevent any mixing. After complete homogenization of all tissue, 500 µL more QIAZOL® reagent was added per sample, followed by a 5-min incubation period to allow for the denaturation of nuclear proteins. Treatment with 140 µL of chloroform per sample, including vigorous shaking for ~ 15 s, 2–3 min of incubation, and centrifugation for 15 min at 12,000 g at 4 °C, was used to achieve phase separation. The upper aqueous phase of each mRNA sample is transferred to a new tube and thoroughly mixed with 525 µL of ethanol for RNA precipitation. Total volumes of this mixture were transferred to a spin column from a miRNeasy® Mini Kit (Qiagen). Further mRNA extraction and purification were performed following the kit manufacturer’s instructions, including the optional on-column DNase digestion with an RNase-free DNase set (Qiagen). Final concentrations of RNA were determined via a Thermo NanoDrop Lite and A260/A280 values for purity confirmation (all ratios were ≥ 2.0).

### Quantitative reverse transcription PCR (RT-qPCR)

First-strand cDNA synthesis was performed via reverse transcription of 200 – 400 ng of total RNA per sample using the SuperScript III Kit First-Strand Synthesis System (Thermo Fisher Scientific), following the manufacturer’s instructions. In a 96-well, hard-shell PCR plate (Bio-Rad, Hercules, CA), 10µL of iTaq Universal SYBR® Green Supermix (Bio-Rad) was mixed with 1 µL of forward and 1 µL reverse primers (Integrated DNA Technologies, Coralville, IA; sequences in Supplementary Table 1) for each respective gene, and 5 µL of MQ water was placed in each well where samples would be amplified. Triplicate amplification reactions were then performed using 3 µL per well of first strand cDNA from each sample on a CFX96 Touch Real-Time PCR Detection System (Bio-Rad) according to the manufacturer’s instructions. MQ water was used in place of cDNA in three wells per triplicate set as a negative control. Amplification of the *Gapdh* gene, a housekeeping gene consistently expressed among all samples, was performed to normalize amplification thresholds and compare expression target gene expression. The level of mRNA expression was evaluated as the fold change relative to *Gapdh* expression. Further analysis compared these relative mRNA expression levels between experimental and control groups. All RT-qPCR results were analyzed by the ΔΔCt method (Livak and Schmittgen 2001).

### Data analysis and statistics

All data were analyzed and graphed using Prism software (GraphPad, v10). Prior to analysis, outliers were identified using the ROUT method (Q = 1%) and removed. Locomotor, TOM ratio, electrophysiological, and qPCR data were analyzed with 2way ANOVAs. The between subjects’ variables were sex (male or female) and group (saline, meth abstinence day 7, or meth abstinence day 28). For time spent interacting during the TOM task, a 3way ANOVA was used for the initial analysis (sex, group, and the within subjects’ variable: object) before decomposing into separate 2way ANOVAs by sex. Significant interactions or main effects of group were followed up with Holm-Šídák’s (behavior), Tukey’s (electrophysiology), or Fisher’s (RT-qPCR) multiple comparisons tests. Different cell numbers within electrophysiology data are due to exclusion of outliers (Q = 1%). Data are presented as mean ± SEM and significance was set at α < 0.05. Complete ANOVA tables are included in the supplement.

## Results

### Sex differences in meth sensitization and temporal order memory during abstinence

Locomotor activity across the experiment was recorded to determine meth’s sensitizing effects, resulting in sex-and group-specific changes in locomotion (Fig. 2A, 2way ANOVA, Sex x Group interaction, F(4,115) = 17.89, p < 0.0001). Holm-Šídák’s post-hoc comparisons revealed that all groups from both sexes had increased activity relative to saline after the first injection of meth (p’s = 0.002–0.0001). Male rats increased locomotion from meth day 1 to day 14 (p = 0.022), an increase that was maintained through abstinence day 7 (p = 0.007). By abstinence day 28, locomotor activity returned to meth day 1 levels. Similarly, female rats increased locomotor activity from meth day 1 to day 14 (p < 0.0001), However, females exhibited an increase in activity from meth day 14 to abstinence day 7 (p = 0.033) indicating an extended sensitization period relative to males. Locomotor activity decreased by abstinence day 28 relative to day 7 (p < 0.0001) but was still higher than meth day 1 (p = 0.0073). Females in all groups, except saline, exhibited greater locomotor activity than males (main effect of sex F(1,115) = 159.3 p < 0.0001). These results demonstrate sex-specific effects in the degree of locomotor sensitization induced by meth in males and females, which extend at least into 28 days of abstinence.

Cognitive function was assessed by performance of the TOM task (Fig. 2B). Time spent interacting with each object (first object, A; second object, B) differed across sex, group, and object (Fig. 2C, 3way ANOVA, Sex x Group x Object interaction, F(2,39) = 7.385, p = 0.0019). Data was separated by sex and analyzed with 2way ANOVAs. For males, only saline subjects spent more time with Object A over Object B (Group x Object interaction, F(2,38) = 8.375, p = 0.0008; and Holm-Šídák’s p = 0.0008). Like the male saline group, female saline subjects also spent more time with Object A (Group x Object interaction, F(2,40) = 8.366, p = 0.0009 and Holm-Šídák’s p = 0.009); however, females also preferred Object A after 28 days of abstinence (p = 0.001).

**Fig. 3:**
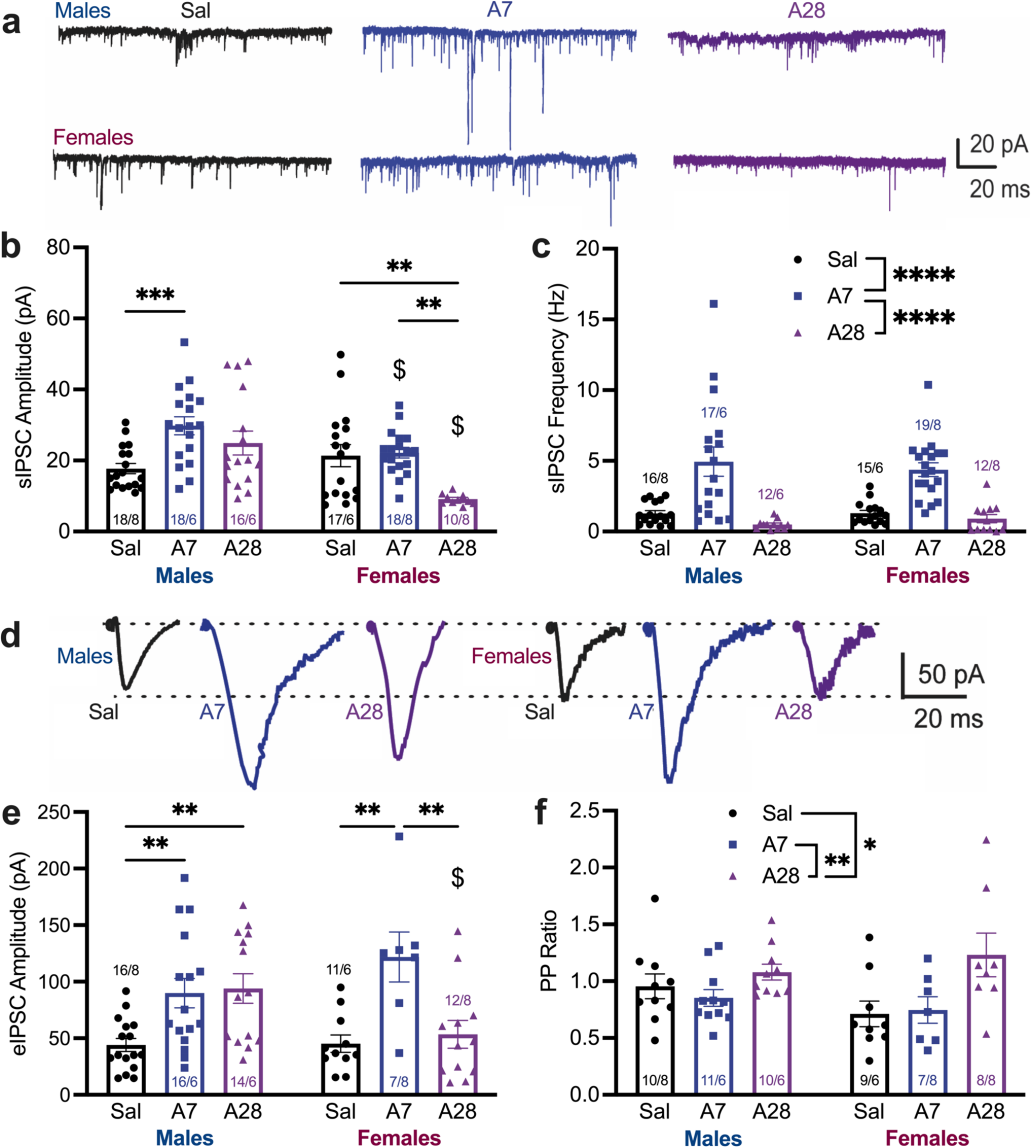
Meth abstinence induces changes in mPFC GABAergic neuron physiology in a sex-specific manner. **A**: Representative traces of sIPSCs from males (top) and females (bottom) in all three groups. **B**: sIPSC amplitude (pA) following saline and short (A7) or prolonged (A27) meth abstinence. sIPSC amplitude was increased in males at A7, but was intermediate (between Sal and A7 levels) at A28. Female sIPSC amplitude was unchanged at A7, but decreased at A28. sIPSC amplitude was lower in females than males at both A7 and A28 (Tukey’s post-hocs; ** p < 0.01, *** p < 0.001, $ p < 0.05 vs males). **C**: sIPSC frequency (Hz) following saline and short (A7) or prolonged (A27) meth abstinence. sIPSC frequency in both sexes was increased at A7 compared to Sal and A28 (Tukey’s post-hocs; **** p < 0.0001). **D**: Representative traces of eIPSCs from males (left) and females (right) in all three groups. **E**: eIPSC amplitude (pA) following saline and short (A7) or prolonged (A27) meth abstinence. eIPSC amplitude was increased in males at A7 and A28. In females, eIPSC amplitude was also increased at A7, but returned to Sal levels at A28. sIPSC amplitude was lower in females than males at A28 only (Tukey’s post-hocs; ** p < 0.01, $ p < 0.05 versus males). **F**: PP ratios following saline and short (A7) or prolonged (A27) meth abstinence. PP ratios were elevated at A28 compared to Sal and A7 (Tukey’s post-hocs; * p < 0.05, ** p < 0.01). Data are shown as mean ± SEM with individual data points (numbers indicate cell *n* / subject *n*). Abbreviations: Sal: Saline, A7: Abstinence Day 7, A28: Abstinence Day 28, sIPSC: Spontaneous inhibitory post-synaptic current, eIPSC: Evoked inhibitory post-synaptic current, PP: paired pulse

Data were also converted to a ratio of time spent with object A and object B. Different patterns of behavior emerged between sexes (Fig. 2D; Sex x Group interaction, F(2,36) = 5.615, p = 0.008). Both sexes had lower TOM indices on abstinence Day 7 (Holm-Šídák’s male p < 0.0001; female p = 0.0003) and in males this was also the case on abstinence day 28 (p < 0.0001). In contrast, females were not different that saline following 28 days of abstinence, but were significantly different from the day 7 group (p = 0.0009). In addition, females had higher TOM ratios than males on day 28 (p = 0.003). To determine that one object engendered a preference over the other and that object interaction was not due to chance exploration all groups were compared to a hypothetical mean of “0” using One Sample t-tests. Both males and females who received saline exhibited a preference for the first object (primacy effect; One sample t-test, Males: t(7) = 6.747, p = 0.0003; Females: t(5) = 6.644, p = 0.0012). After 7 days of abstinence from meth, both males and females had an interaction preference to the second object (recency effect; One sample t-test, Males: t(5) = 2.797, p = 0.0381; Females: t(7) = 2.662, p = 0.0324). Following 28 days of abstinence, males did not exhibit a preference for either object, however females did demonstrate a preference for the first object (One sample t-test, t(7) = 2.931, p = 0.0220). In order to investigate the mechanisms underlying this sex difference, we tested a group of OVX females (supplementary data). Notably, in OVX females, TOM performance was disrupted across all 3 groups, but daily estradiol (E2) replacement restored TOM in all 3 groups (Fig. S1, see Supplement for details).

### Sex differences in GABAergic transmission during meth abstinence

To investigate changes in E/I balance in male and female rats following short (7 days) or prolonged (28 days) abstinence, we used whole-cell mode patch-clamp recordings of deep-layer (layer V–VI) pyramidal neurons (PNs) in acute slice preparations (see methods). Representative traces of sIPSCs from males and females are shown in Fig. 3A. Following meth sensitization, there were sex- and group-specific changes in sIPSCs amplitude during abstinence (Fig. 3B, 2way ANOVA, Sex x Group interaction, F(2,91) = 7.522, p = 0.0009). In males, sIPSCs amplitude was elevated on abstinence day 7 compared to saline controls (Tukey’s p = 0.001). After prolonged abstinence, sIPSCs amplitude in males was not significantly different from saline or abstinence day 7. In females, sIPSCs amplitude was unchanged after 7 days of abstinence, but after 28 days it was significantly decreased relative to saline (p = 0.006) or abstinence day 7 (p = 0.003). Despite having similar sIPSCs amplitude in saline controls, females had lower amplitudes at both 7 (p = 0.02) and 28 (p = 0.0001) days of abstinence compared to males. In contrast, changes in sIPSCs frequency were similar between males and females (Fig. 3C, 2way ANOVA, main effect of Group, F(2,85) = 30.67, p < 0.0001). Following 7 days of abstinence, sIPSCs frequency increased relative to saline (p = 0.0001) and returned to saline values by day 28 (abstinence day 7 compared to 28, p = 0.0001).

Representative traces of eIPSCs from males and females are shown in Fig. 2D.There were sex- and group-specific changes in eIPSCs amplitude during abstinence (Fig. 3E, 2way ANOVA, Sex x Group interaction, F(2,70) = 4.180, p = 0.0193). Males had increases relative to saline in eIPSCs amplitude following 7 days (Tukey’s p = 0.0087) and 28 (p = 0.006) days of abstinence from meth. Females also had increased amplitude at day 7 relative to saline (p = 0.0011), but eIPSCs returned to a value consistent with saline by day 28 (abstinence day 7 compared to 28, p = 0.0032). Increases in eIPSCs amplitude were similar between the sexes, but females had lower amplitudes at day 28 compared to males (p = 0.018). Another measure of synaptic activity, PP ratio, differed by group but not sex (Fig. 3F, 2way ANOVA, main effect of Group, F(2,49) = 5.952, p = 0.0049). This effect was driven by elevated PP ratios on abstinence day 28 compared to saline (p = 0.016) and abstinence day 7 (p = 0.009) groups. These results demonstrate that meth elicits sex-dependent changes in pre- and post-synaptic GABAergic activity in the mPFC.

### Meth decreases the mRNA expression of prefrontal GABAA receptors and transporter

**Fig. 4:**
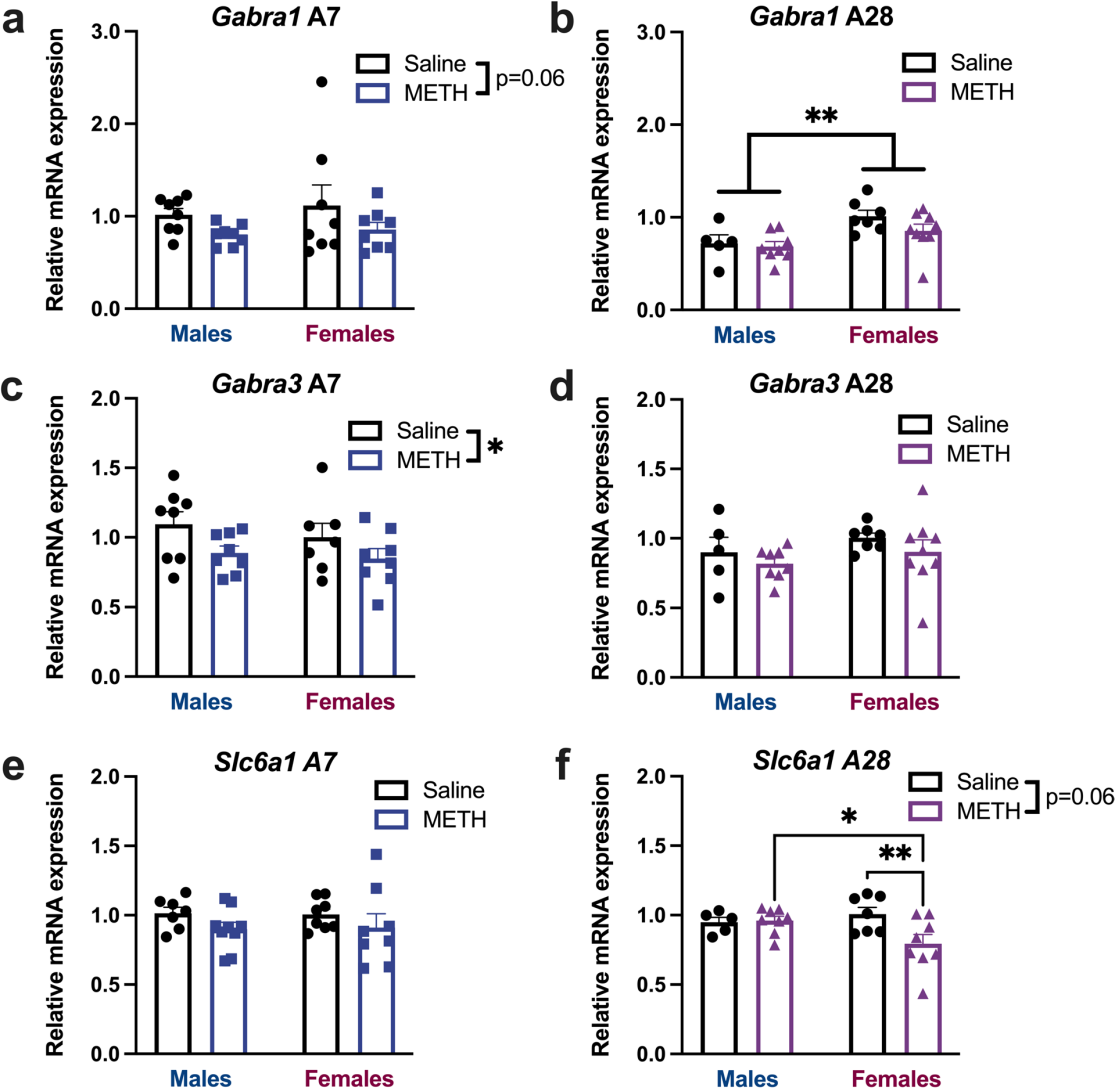
Meth abstinence changes GABA-associated mRNA expression in the mPFC. **A**: *Gabra1* relative mRNA expression after 7 days of abstinence from meth. Meth exposure resulted in a trend towards decreases in *Gabra1* (p = 0.06). **B**: *Gabra1* relative mRNA expression after 28 days of abstinence from meth. Females had greater *Gabra1* mRNA than males, regardless of meth exposure (** p < 0.01). **C**: *Gabra3* relative mRNA expression after 7 days of abstinence from meth. Meth exposure decreased in *Gabra3* mRNA in both sexes (* p < 0.05). **D**: *Gabra3* relative mRNA expression after 28 days of abstinence from meth. There were no differences in *Gabra3* mRNA at this timepoint. **E**: *Slc6a1* relative mRNA expression after 7 days of abstinence from meth. There were no differences in *Slc6a1* mRNA at this timepoint. **F**: *Slc6a1* relative mRNA expression after 28 days of abstinence from meth. Meth decreased *Slc6a1* mRNA in females relative to meth males and saline females (Fisher’s LSD post-hocs; * p < 0.05, ** p < 0.01). Data are shown as mean ± SEM with individual data points (numbers indicate cell *n* / subject *n*). Abbreviations: Sal: Saline, A7: Abstinence Day 7, A28: Abstinence Day 28, *Gabra1*, GABA_A_ receptor subunit α1; *Gabra3*, GABA_A_ receptor subunit α3; *Slc6a1*, GABA transporter 1

Given our previous (Armenta-Resendiz et al. 2022) and present findings showing that meth affects pre- and post-synaptic GABAergic activity, we measured mRNA expression of GABA_A_R and GAT-1—following meth sensitization in an independent group of male and female rats. We selected the GABA_A_R subunits α1 (*Gabra1*) and α3 (*Gabra3*), since these subunits form part of most postsynaptic GABA_A_Rs that regulate phasic inhibition in the cortex (Wearne et al. 2016; Barker et al. 2007; Sente et al. 2022), and GAT1 (*Slc6a1*). Relative mRNA levels for each marker at abstinence day 7 and day 28 were compared using separate 2way ANOVAs (Fig. 4). At abstinence day 7, levels of *Gabra1* mRNA trended lower in response to meth exposure (Fig. 4A, main effect of Meth, F(1,28) = 3.723, p = 0.0638). At abstinence day 28, females had higher *Gabra1* mRNA levels than males (Fig. 4B, main effect of Sex, F(1,25) = 10.45, p = 0.0034). *Gabra3* mRNA was lowered following 7 days of meth abstinence (Fig. 4C, main effect of meth, F(1,27) = 5.084, p = 0.0325), but meth-induced changes did not persist following 28 days of abstinence (Fig. 4D). There were no differences in *Slc6a1* mRNA following 7 days of abstinence (Fig. 4E), however meth decreased Slc6a1 in females only on abstinence day 28 (Fig. 4F, Sex x Meth interaction, F(1,24) = 4.647, p = 0.0413). The main effect of meth also approached significance (F(1,24) = 3.747, p = 0.0648). Specifically, 28 days after meth exposure, *Slc6a1* mRNA was significantly decreased in meth females compared to saline females and meth males (Fisher’s uncorrected LSD post-hoc, saline females versus meth females p = 0.0056; meth males versus meth females p = 0.0216). In summary, these results show a meth-induced reduction in the expression of GABAergic receptor subunit mRNAs (*Gabra1* and* Gabra3*) at abstinence day 7 that normalizes by day 28. Presynaptically, meth does not impact *Slc6a1* mRNA levels following 7 days of abstinence, however, there is a sex-specific reduction of this marker in females at day 28. Regardless of meth exposure, females had higher *Gabra1* mRNA than males at day 28.

## Discussion

We report here that repeated meth administration elicits significant behavioral and physiological changes according to subject sex and meth abstinence period. Specifically, we report increased meth-induced locomotor activity and persistent sensitization in females, similar changes in meth-induced memory processing with greater recovery of function in females, and recovery of GABAergic transmission depending on the duration of abstinence and sex. Interestingly, recovery of memory deficits and changes in GABA transmission do map, to a certain extent, onto changes in GABA_A_R expression. However, unique decreases in *Slc6a1* mRNA (GAT-1) exist in meth exposed females after 28 days of abstinence.

### Sex differences in meth sensitization and temporal order memory during abstinence

During the meth administration period, females had higher distance traveled scores in response to an acute injection (Milesi-Halle et al. 2007; Ohia-Nmoko et al. 2017) and after a 14-day sensitization regimen (Wearne et al. 2016). Unique to our study, testing at multiple timepoints demonstrated that a sensitized response is still evident in females even after 28 days of meth abstinence. Albeit, this response is waning, but activity was still elevated relative to the first meth injection. The persistence of a sensitized response following 28 days is important because at this timepoint we also found recovery of meth induced memory impairment and decreases in frequency of sIPSCs and amplitude of eIPSCs in females but not males, suggesting a restoration of levels of GABA transmission in females. Where males no longer expressed a sensitized response, they still exhibited memory impairments and increased amplitude of s and eIPSCs. These results suggest that the changes in GABAergic transmission in the mPFC and concurrent cognitive deficits may be independent of the mechanism mediating meth locomotor sensitization. How this sex specific pattern of findings relates to meth induced hypofrontality and cognitive function remains to be completely elucidated.

Our previous findings have shown that volitional and non-volitional methamphetamine exposure results in memory deficits including object recognition (Hopkins et al. 2023; Peters et al. 2018, Peter et al. 2016;  Reichel et al. 2011), object-in-place (Bernheim et al. 2016; Reichel et al. 2014), and TOM (Armenta-Resendiz et al. 2022). Importantly, meth causes deficits in object recognition and object-in-place recognition memory in both sexes (Hopkins et al. 2023; Reichel et al. 2014), but this is the first demonstration of sex differences in recovery of episodic memory function. The aforementioned tasks encompass different elements of episodic memory that are mediated by cortical processes. Temporal order tasks evaluate the “when” component of the memory process. In the TOM task rats are expected to show a preference for an earlier object (without training or reward) because that object has not been recently experienced and has therefore regained some aspects of “novelty” (Ennaceur 2010). Preference for items sampled first is in alignment with the psychological notion of the “primacy effect” whereas items sampled closer in time with the “recency effect”. Indeed, object sampling of saline animals (both sexes) demonstrated the primacy effect; whereas meth experience changed object sampling to a recency effect after 7 days of abstinence. This is noteworthy because in an object recognition or object-in-place task, object sampling is typically at chance, or equal, performance after meth (Hopkins et al. 2023; Reichel et al. 2011, 2014), indicating no preferential exploration of either object. Yet, here meth experience shifted TOM to a preference for the object experienced most recently. This finding begs the question: Does the recency bias in meth rats after seven days of abstinence result from a deficit in one aspect of TOM while simultaneously preserving another? The answer to this question is beyond the scope of this set of experiments and would require systematic exploration. However, meth impacts multiple cognitive and memory processes that rely on intact function of the prefrontal cortex, as well as the perirhinal cortex and hippocampus (Bernheim et al. 2016; Hopkins et al. 2023; Peters et al. 2018, 2016; Reichel et al. 2011, 2014; Barker and Warburton 2020). We suggest that this recency effect results from a dissociation between cognitive processes involving timing and memory. Indeed, temporal separation and object recognition are suggested to be independent processes (Barker et al. 2019) mediated by distinct circuits involving the prefrontal cortex, perirhinal cortex, and hippocampus (Barker and Warburton 2020).

Even more intriguing is the recovery of the primacy effect in females after 28 days of abstinence, but not in males. On this day, males exhibited no preference for either object, exploring both at chance levels. While beyond the scope of this project, we propose that female rats have protective elements that allow them to recover faster from the changes in cognitive function and inhibitory transmission in mPFC induced by meth. One such difference is estrogen signaling, which has been reported to have neuroprotective effects against meth exposure (Culmsee et al. 2011; Gao and Dluzen, 2001) mediated by a number of factors, including antioxidant effects (Sawada et al. 1998), alterations of dopamine transporter function (Chavez et al. 2010;  D'Astous et al. 2004) or potentiation of excitatory neurotransmission (Potier et al. 2016; Srivastava et al. 2013). Here, we provide supplemental evidence (Figure S1) for a contribution of estrogen signaling to sex differences in TOM recovery after meth. OVX impairs novel object and object-in-place recognition memory in female rats (Wallace et al. 2006), which we have extended to TOM in this report (Figure S1). Estradiol administered to OVX females improves many cognitive processes (reviewed in 63). Importantly, here, daily estradiol given to both saline- and meth-treated OVX female rats restored TOM task performance (primacy effect, Figure S1). This supplemental behavioral data supports a role for estrogen signaling in recovery of meth-induced TOM deficits that should be explored in future studies.

### Sex differences in GABAergic transmission during meth abstinence

In male rats, meth administration followed by 7 days of abstinence increased GABAergic transmission in the mPFC via pre- and post-synaptic increases in the frequency and amplitude of sIPSCs and eIPSCs (Armenta-Resendiz et al. 2022). We posit that this increase in inhibitory transmission alters E/I balance in the mPFC, resulting in hypofrontality and concomitant deficits in TOM. The present results (Fig. 3) recapitulate our previous findings in males and show that following short-term abstinence (7 days), both males and females undergo changes in both presynaptic (increased frequency of sIPSCs) and postsynaptic (increased amplitude of eIPSCs) function. Furthermore, we have previously shown that chemogenetic manipulation of parvalbumin-positive interneuron activity in meth rats ameliorates meth-evoked deficits in TOM, suggesting a link between increased GABAergic activity and TOM deficits (Armenta-Resendiz et al. 2022).

After a longer period of abstinence (28 days), we find enduring post-synaptic changes in the male group, however, the female rats show restoration of the sIPSC frequency and eIPSC amplitude. These results demonstrate that meth elicits different alterations in GABA neurotransmission in the mPFC, depending on the sex of the animals and the duration of abstinence. Furthermore, these electrophysiological changes in the mPFC correspond with the time course and sex-specificity of TOM behavioral deficits. Interestingly, in a supplemental experiment, sIPSC and eIPSC amplitudes in the mPFC were decreased by daily estradiol administration in both saline and A7 meth-exposed OVX females (Figure S1). This is consistent with similar decreases in sIPSC and eIPSC amplitudes observed on abstinence day 28 in gonadally-intact females (Fig. 3). As in the main text, these changes also correspond to behavioral restorations in TOM performance (Figure S1). This provides further, albeit indirect, evidence of a role for estrogen signaling to impact GABAergic neuron function in the mPFC to enhance TOM performance. While beyond the scope of this study, future studies should examine the mechanisms by which estrogen signaling alters inhibitory neuron function in the mPFC.

**Fig. 5:**
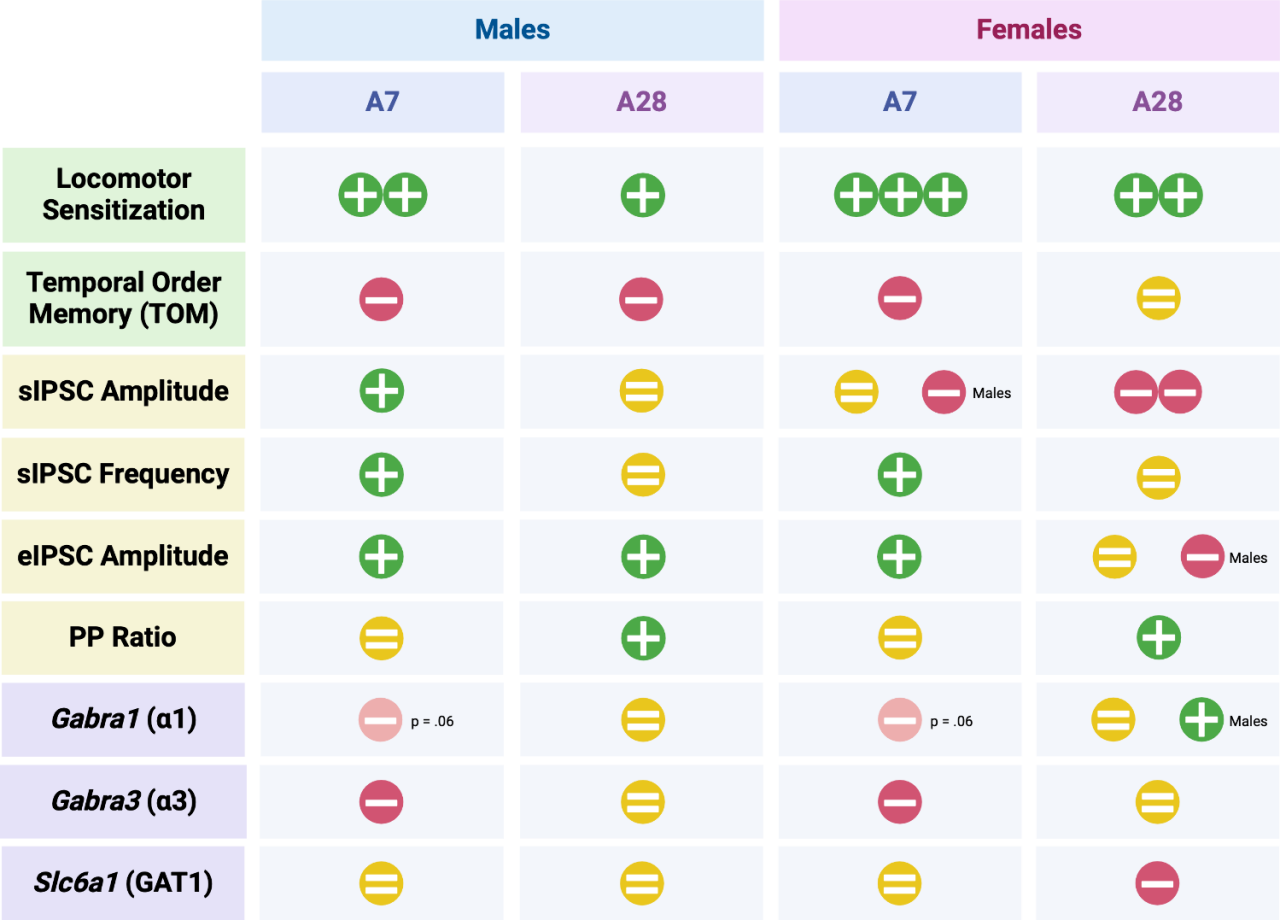
Summary of findings. Changes in the outcome measured (rows) by sex (collapsed columns) and abstinence duration (individual columns). All changes are indicated relative to saline controls, unless otherwise specified (some comparisons between males and females indicated). Direction of change relative to saline controls is indicated as follows: green + : increase; red –: decrease; yellow = : no change. Number of symbols does not reflect the magnitude of the difference, except in row 1 (locomotor sensitization)

Given the meth-mediated changes in pre- and post-synaptic markers of GABAergic transmission, we investigated the mRNA levels of two of the constitutive subunits of the GABA_A_ post-synaptic receptor, α1 and α3 (*Gabra1* and *Gabra3*), and the transporter, GAT1 (*Slc6a1*, Fig. 4). GABA_A_Rs mediate phasic inhibition (Ghit et al. 2021). After short abstinence, meth elicited significant decreases in *Gabra3* (approaching significance for *Gabra1*) mRNA in both sexes. These results are consistent with the electrophysiological findings that after 7 days of abstinence, both males and females exhibit increases in post-synaptic markers of GABAergic transmission in the mPFC (i.e., increased amplitude of sIPSCs and eIPSCs) and correlate with the deficits in TOM. However, the lack of change in *Slc6a1* is inconsistent with presynaptic increases in sIPSC frequency on abstinence day 7. Since the IPSCs are recorded selectively in pyramidal cells, it is possible that the mRNA analyses mainly represent levels of GAT1 on other cells, since interneurons are so few in the PFC (Fig. 5).

Contrary to our findings, some studies report that after repeated meth exposure, GABA_A_R subunit α1 and GAT1 are significantly increased (Wearne et al. 2016; however, those experiments only administered meth for 8 days and the animals were tested following a meth challenge dose of 1 mg/kg of meth 60 min before decapitation, in contrast to our mRNA analyses that were conducted in a drug-free state.

After a longer abstinence period (28 days), *Gabra1* and *Gabra3* mRNA levels in the mPFC of meth-exposed subjects are not different from saline controls. Interestingly, at this time point, females have recovered from the behavioral and electrophysiological changes. Several differences could contribute to this functional restoration that are not reflected by *Gabra1* or *Gabra3* mRNA levels. For example, *Slc6a1* mRNA was lower in meth females at A28, indicating pre-synaptic changes in regulation of GABAergic signaling. Additionally, glutamatergic or other GABAergic adaptations not examined herein could also underlie recovery. Indeed, we have previously reported that following meth-SA, female rats exhibit increases in the amplitude of NMDA currents, mediated by an increase in GluN2B-lacking NMDA receptors (Pena-Bravo et al. 2019) suggesting at least one possible mechanism underlying female recovering of synaptic changes and temporal order memory. And we cannot rule-out that deficits in cognitive function are mediated by other brain regions, beside the mPFC. Interestingly, females, regardless of meth or saline, had greater *Gabra1* mRNA than males at A28. We did not identify a clear functional significance, if any, for this difference in the present study.

One accepted means of modifying the efficacy of synaptic transmission under conditions of neurotransmitter saturation is to change the number and/or sensitivity of post-synaptic receptors. Indeed, Lyons and colleagues (2013) have shown that persistent exposure (48 h) of brain neurons in primary culture to GABA, results in a 30% decrease in the levels of mRNA encoding the α1, β2S, and γ1 GABA_A_R subunit isoforms. Furthermore, other studies have documented that occupancy of GABA_A_Rs by GABA induces a down-regulation in receptor number (Friedman et al. 1996; Roca et al. 1990). Interestingly, several papers report changes in the GABA_A_R subunit composition and decreased GABA function after long-term exposure to GABA_A_R agonists (Miller et al. 1988; Belelli et al. 2002. Thus, our results measuring mRNA levels of constitutive GABA_A_R subunits and GAT1 supports the hypothesis that repeated meth increases GABAergic transmission following short abstinence in male and female rats.

### Enhanced recovery of function in females

Taken together, our results show that following short abstinence, male and female rats exhibit enhanced GABAergic activity in the mPFC that may mediate the deficits in elements of episodic memory related to temporal processes. Furthermore, we found that females recover from the meth-mediated cognitive deficits and increases in GABAergic transmission in the mPFC by 28 days of abstinence, whereas males do not. We have previously reported that volitional meth increased mPFC-evoked excitatory currents in female rats (Pena-Bravo et al. 2019). This increase in evoked glutamate was correlated with increases in NMDA currents (Pena-Bravo et al. 2019) supporting the suggestion that estrogens (present in freely cycling females) may potentiate excitatory transmission, resulting in a reestablishment of the E/I balance in the mPFC. Correct E/I balance is necessary for optimal cognitive performance and E/I ratio alterations in the frontal cortex are thought to contribute to various neuropsychiatric disorders (Lopatina et al. 2019; Foss-Feig et al. 2017, including substance use disorders (Sohal and Rubenstein 2019; Goldstein et al. 2002; Volkow et al. 1991; Volkow et al. 2009; Bolla et al. 2003). Furthermore, we report that estradiol given to OVX females decreases inhibitory sIPSC and eIPSC amplitude regardless of saline or meth condition (Figure S1). The full extent of estradiol on E/I ratio has not been fully elucidated, but remains a viable option given the role of estrogens neuroprotective effects (Culmsee et al. 2011; Gao and Dluzen 2001; Sawada et al. 1998; Chavez et al. 2010; D'Astous et al. 2004; Bosse et al. 1997. Future studies aimed at monitoring the peripheral and central levels of circulating ovarian hormones in female rats during the performance of episodic memory tasks will be shed light on this issue.

## Conclusions

Overall, our previous results showing that meth induced deficits in episodic memory performance and changes in pre- and post-synaptic GABA transmission (Armneta-Resendiz et al. 2022) is extended to female rats on abstinence day 7. Importantly, we demonstrated that females recover from these changes by abstinence day 28, even though they are still displaying evidence of meth sensitization at that time point. Males, in contrast, still exhibited memory impairments and changes in E/I ratio, but lack of sensitized effects. As such, we have demonstrated that GABAergic transmission in the PFC and concurrent cognitive deficits are independent of the mechanism mediating meth locomotor sensitization. How this sex specific pattern of findings relates to meth-induced hypofrontality and cognitive function remains to be completely elucidated. Estradiol may have a role in mediating recovery of function in females contributing to improved memory and restored E/I ratio indicating that sex and abstinence duration are important variables to consider in meth-induced hypofrontality.

## Supplementary Information

Below is the link to the electronic supplementary material.

Supplementary file1 (DOCX 1101 KB)

Supplementary file2 (XLSX 76 KB)
