# Supplementary material for: Sex differences in behavior, cognitive, and physiological recovery following methamphetamine administration
Source: Psychopharmacology (Berl). 2024 Jul 2;241(11):2331–45. doi: 10.1007/s00213-024-06638-1 (PMC11513735; doi:10.1007/s00213-024-06638-1)
Supplement: Supplementary file 1 — Supplementary file1 (DOCX 1101 KB) [file 213_2024_6638_MOESM1_ESM.docx]

*Psychopharmacology* Supplement for:

Sex differences in behavior, cognitive, and physiological recovery following methamphetamine administration

Monserrat Armenta-Resendiz, PhD*****, Jordan S. Carter, PhD*****, Zachariah Hunter, BS, Makoto Taniguchi, PhD, Carmela M. Reichel, PhD, Antonieta Lavin, PhD

Department of Neuroscience, Medical University of South Carolina, Charleston, SC 29425

*Denotes equal authorship

**Corresponding Author**:

Antonieta Lavin

Department of Neuroscience

Medical University of South Carolina

Email: lavina@musc.edu

Phone: 843-792-6799

Address: 173 Ashley Ave

BSB 403 (MSC: 510)

Charleston, SC 29425

**Supplementary Experiment**: **Ovariectomized Females and Estradiol Replacement**

**Methods:**

**OVX females underwent meth administration, locomotor activity, TOM assessment, and electrophysiologic recordings as described in the main text. A subset of females were treated with daily estradiol (E2) injections (**10 μg/kg, i.p.). The E2 dose was selected based on the work of Doncheck et al. (2018 and 2021) where they reported that these dose mimics circulating proestrus levels. Rats were administered with E2, starting 10 days before the meth treatment and during all of the meth treatment (14 days). This protocol was established based on previous reports regarding the estradiol levels post-ovariectomy (de Chaves et al., 2009; Moiety et al., 2015).

**Data analysis was also as described in the main text, except between subjects’ variables for the 2way ANOVAs were hormone status (OVX or OVX+E2) and group (saline, meth abstinence day 7, or meth abstinence day 28). Complete ANOVA tables are shown in Table S6.**

**Results:**

**Locomotor activity across the experiment was recorded to determine meth’s sensitizing effects, resulting in E2- and group-specific changes in locomotion (Fig. S1a, 2way ANOVA, E2 x Group interaction, F(3,43) = 5.964, p = 0.0017). Compared to saline, OVX females had increased locomotor activity only on meth day 14 (p < 0.0001). OVX female locomotion on meth day 14 was also greater than their activity on meth day 1 (p = 0.001) and abstinence day 28 (p = 0.010). OVX females who received E2 had greater locomotor activity compared to saline on all other days (meth day 1, meth day 14, and abstinence day 28; p’s ≤ 0.0003). Locomotor activity between saline groups did not differ, but OVX+E2 females were more active on meth day 1 (p < 0.0001) and abstinence day 28 (p = 0.0029) compared to OVX females. There were no differences on meth day 14.**

**Subjects had cognitive function assessed by a TOM task (Fig. S1b). Time spent interacting with each object (first object, A; second object, B) differed by E2 and object (Fig. S1c, 3way ANOVA, E2 x Object interaction, F(1,39) = 8.194, p = 0.0067). Data was separated by E2 and analyzed with 2way ANOVAs. For OVX females, there were no differences in time spent between the objects for any group. In contrast, OVX+E2 female subjects from all three groups spent more time with Object A (2way ANOVA, main effect of Object, F(1,38) = 43.47, p < 0.0001).**

**TOM performance can also be indexed by the TOM discrimination ratio (Fig. S1d). Analysis of the TOM ratio revealed effects of E2 (2way ANOVA, main effect of E2, F[1,38] = 7.851, p = 0.0079) such that OVX+E2 females in the saline and 7 day abstinence groups had greater recognition indices compared to OVX females (p’s = 0.0476). As a whole, saline OVX females do not have a preference for either object, however there is a large bimodal distribution with some subjects preferring Object A and some preferring Object B. After 7 days of abstinence from meth, OVX females exhibit a preference for the second object (recency effect; One sample t-test, t(7) = 2.632, p = 0.0338), which by 28 days of abstinence shifts back to no preference. In contrast, OVX+E2 females exhibit a preference for the first object in all three groups (primacy effect; One sample t-test, Sal: t(9) = 10.21, p < 0.0001; A7: t(7) = 4.420, p < 0.0031; A28: t(2) = 8.510, p = 0.0135).**

OVX and OVX+E2 females underwent analysis of GABAergic mPFC transmission following the TOM task (only Sal and A7 data shown; measurements from A28 in OVX+E2 did not yield any results). sIPSC amplitude was elevated in OVX females relative to OVX+E2 (Fig. S1e, 2way ANOVA, main effect of E2, F(1,27) = 7.483, * p < 0.0107). Similarly, eIPSC amplitude was elevated in OVX females compared to OVX+E2 (Fig. S1f, 2way ANOVA, main effect of E2, F(1,30) = 7.966, ** p = 0.0084).

**Supplementary References**:

Doncheck EM, Urbanik LA, DeBaker MC, Barron LM, Liddiard GT, Tuscher JJ, Frick KM, Hillard CJ, Mantsch JR. 17β-Estradiol Potentiates the Reinstatement of Cocaine Seeking in Female Rats: Role of the Prelimbic Prefrontal Cortex and Cannabinoid Type-1 Receptors Neuropsychopharmacology . 2018 Mar;43(4):781-790.  doi: 10.1038/npp.2017.170.

Doncheck EM, Anderson EM, Konrath CD, Liddiard GT, DeBaker MC, Urbanik LA, Hearing MC, Mantsch JR.  [Estradiol Regulation of the Prelimbic Cortex and the Reinstatement of Cocaine Seeking in Female Rats.](https://pubmed.ncbi.nlm.nih.gov/33879537/) J Neurosci. 2021 Jun 16;41(24):5303-5314. doi: 10.1523/JNEUROSCI.3086-20.2021.

de Chaves G, Moretti M, Castro AA, Dagostin W, da Silva GG, Boeck CR, Quevedo J, Gavioli EC. [Effects of long-term ovariectomy on anxiety and behavioral despair in rats.](https://pubmed.ncbi.nlm.nih.gov/19321148/) *Physiol Behav.* 2009 Jun 22;97(3-4):420-5. doi: 10.1016/j.physbeh.2009.03.016.

Moiety FM, Salem HA, Mehanna RA, Abdel-Ghany BS. Comparative study on induction and effects of surgical menopause in a female rat model: a prospective case control study. *Int J Clin Exp Med.* 2015 Jun 15;8(6):9403-11. eCollection 2015.

**Supplementary Figure**:

**
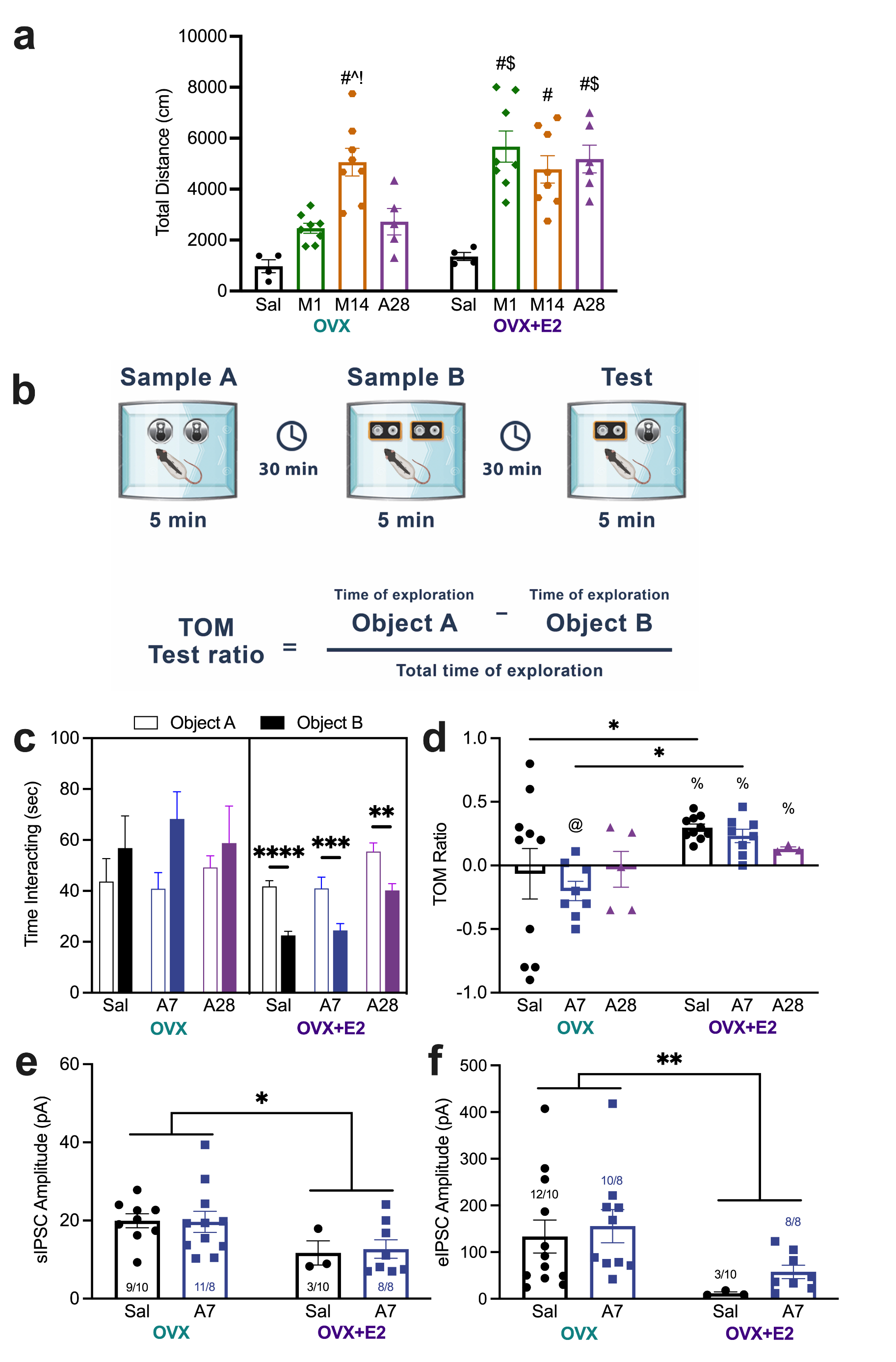
**

**Fig. S1** Estradiol replacement in OVX females prolongs meth-induced locomotor sensitization, protects temporal order memory performance, and alters GABAergic neuron physiology in the mPFC

a: Distance traveled (cm) following meth exposure. OVX and OVX+E2 females increase locomotor activity following meth exposure, but OVX+E2 females sensitize earlier and maintain the sensitizing effect after 28 days of abstinence (Holm-Šídák’s post-hocs: # p < 0.05 vs saline; $ p < 0.05 vs OVX; ^ p < 0.05 vs M1, ! p < 0.05 vs A28).

b: Schematic of TOM task and calculation of TOM ratio.

c: Time interacting (sec) with Object A (first object) and Object B (second object) during TOM test. OVX females spend similar amounts of time with either object, regardless of saline or meth exposure. OVX+E2 females spent more time with Object A after saline and abstinence from meth (Holm-Šídák’s post-hocs; ** p < 0.01, *** p <0.001, **** p < 0.0001).

d: TOM discrimination ratios. OVX females receiving saline on A28 displayed no preference for either object, though there was significant variation in the saline group. On A7, OVX females preferred Object B (@, p < 0.05 preference for Object B vs chance). OVX+E2 females preferred Object A in all three groups (%, p < 0.05 preference for Object A vs chance). E2 prevented both OVX- and meth-induced disruptions in TOM (Holm-Šídák’s post-hocs; * p < 0.05).

e: sIPSC amplitudes (pA) following saline and short (A7) meth abstinence. OVX females, regardless of group, had higher sIPSC amplitude than OVX+E2 females (main effect of E2, * p < 0.05).

f: eIPSC amplitudes (pA) following saline and short (A7) meth abstinence. OVX females, regardless of group, had higher eIPSC amplitude than OVX+E2 females (main effect of E2, ** p < 0.01).

Data are shown as mean ±SEM with individual data points (numbers indicate cell *n* / subject *n*). Abbreviations: Sal: Saline, M1: Meth Day 1, M14: Meth Day 14; A7: Abstinence Day 7; A28: Abstinence Day 28, OVX: Ovariectomy, E2: Estradiol, TOM: Temporal Order Memory, sIPSC: Spontaneous inhibitory post-synaptic current, eIPSC: Evoked inhibitory post-synaptic current

**Supplementary Tables**:

**Table S1**. Primers for RT-qPCR.

| ***Gene* / Protein** | **Forward** | **Reverse** |
| --- | --- | --- |
| ***GAPDH* / GAPDH** |  |  |
| ***SLC6A1* / GAT1** | GCGCAACATGCACCAAATGACA | AGACCACCTTTCCAGTCCATCCAA |
| ***GABRA1* / GABA_A_R α1** | TGTCTTTGGAGTGACGACCGTTCT | ACACGAAGGCATAGCACACTGCAA |
| ***GABRA3* / GABA_A_R α3** | TCTGGATGGCTATGACAACCGACT | ACTTCAGTCACTGCATCTCCAAGC |

**Table S2**. Key resources table.

| ***Software*** | | |
| --- | --- | --- |
| Axograph ® | John Clements |  |
| MiniAnalysis ® | Synaptosoft |  |
| Prism (v.10) | GraphPad |  |
| ***Drugs*** | | |
| Methamphetamine | NIDA Drug Supply |  |
| β-Estradiol water soluble | Millipore Sigma | Catalog No. E4389 |
| ***Reagents*** | | |
| RNAse*Zap* ® | Invitrogen |  |
| QIAZOL ® Lysis Reagent | Qiagen |  |
| miRNeasy ® Mini Kit | Qiagen |  |
| RNase-free DNase | Qiagen |  |
| SuperScript III First-Strand Synthesis Kit | Thermo Fisher |  |
| iTaq Universal SYBR ® Green Supermix | Bio-Rad |  |
| Primers | Integrate DNA Technologies |  |

**Tables S3-S6** (complete ANOVA results) can be found in the supplementary Excel file.
